# Supplementary figures and images for: Seroepidemiology of SARS-CoV-2 in a cohort of pregnant women and their infants in Uganda and Malawi
Source: PLoS One. 2024 Mar 1;19(3):e0290913. doi: 10.1371/journal.pone.0290913 (PMC10906847; doi:10.1371/journal.pone.0290913)

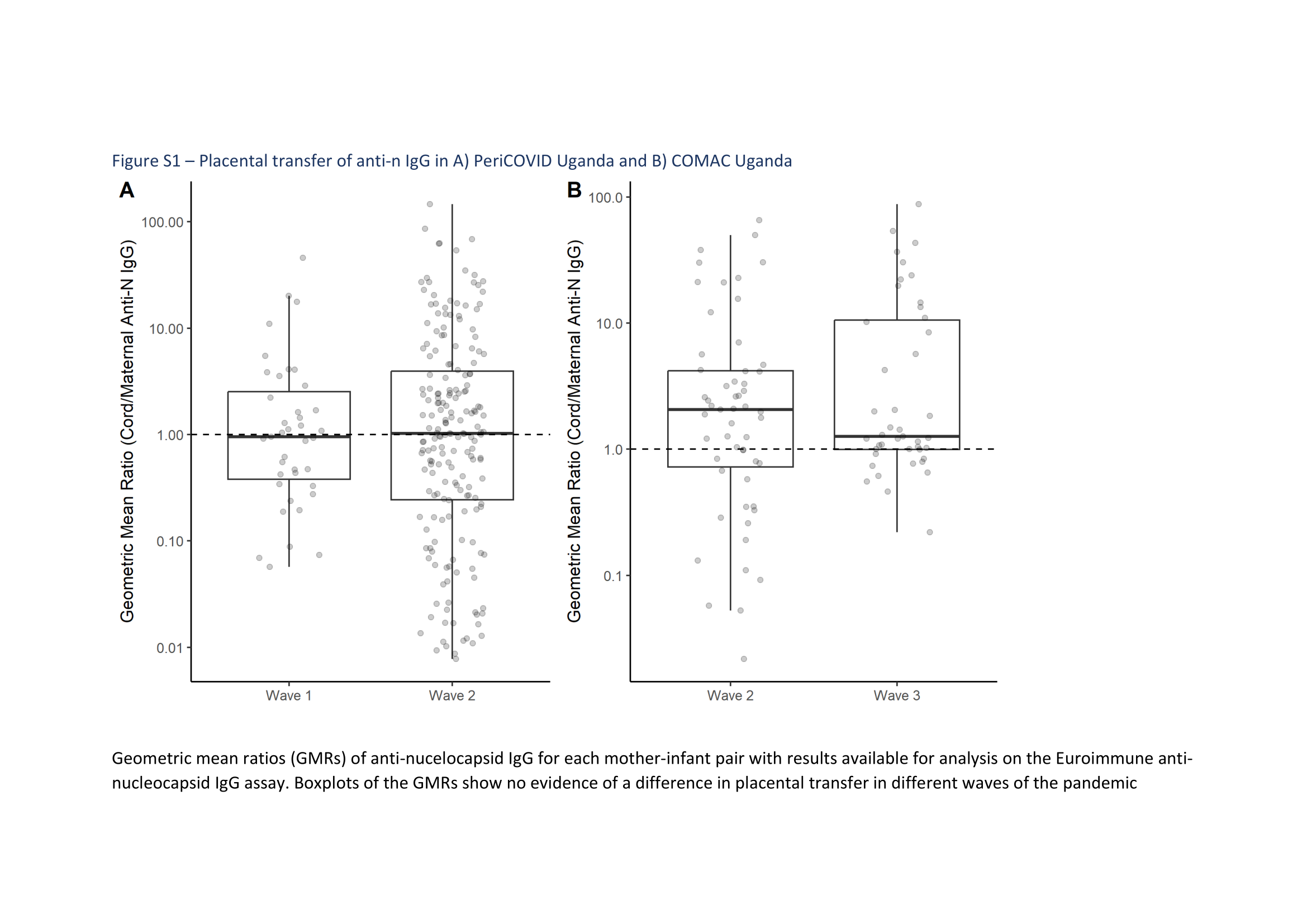

Supplement: S1 Fig — Placental transfer of anti-n IgG in A) PeriCOVID Uganda and B) COMAC Uganda. Geometric mean ratios (GMRs) of anti-nucelocapsid IgG for each mother-infant pair with results available for analysis on the Euroimmune anti-nucleocapsid IgG assay. Boxplots of the GMRs show no evidence of a difference in placental transfer in different waves of the pandemic. (TIF) [file pone.0290913.s001.tif]

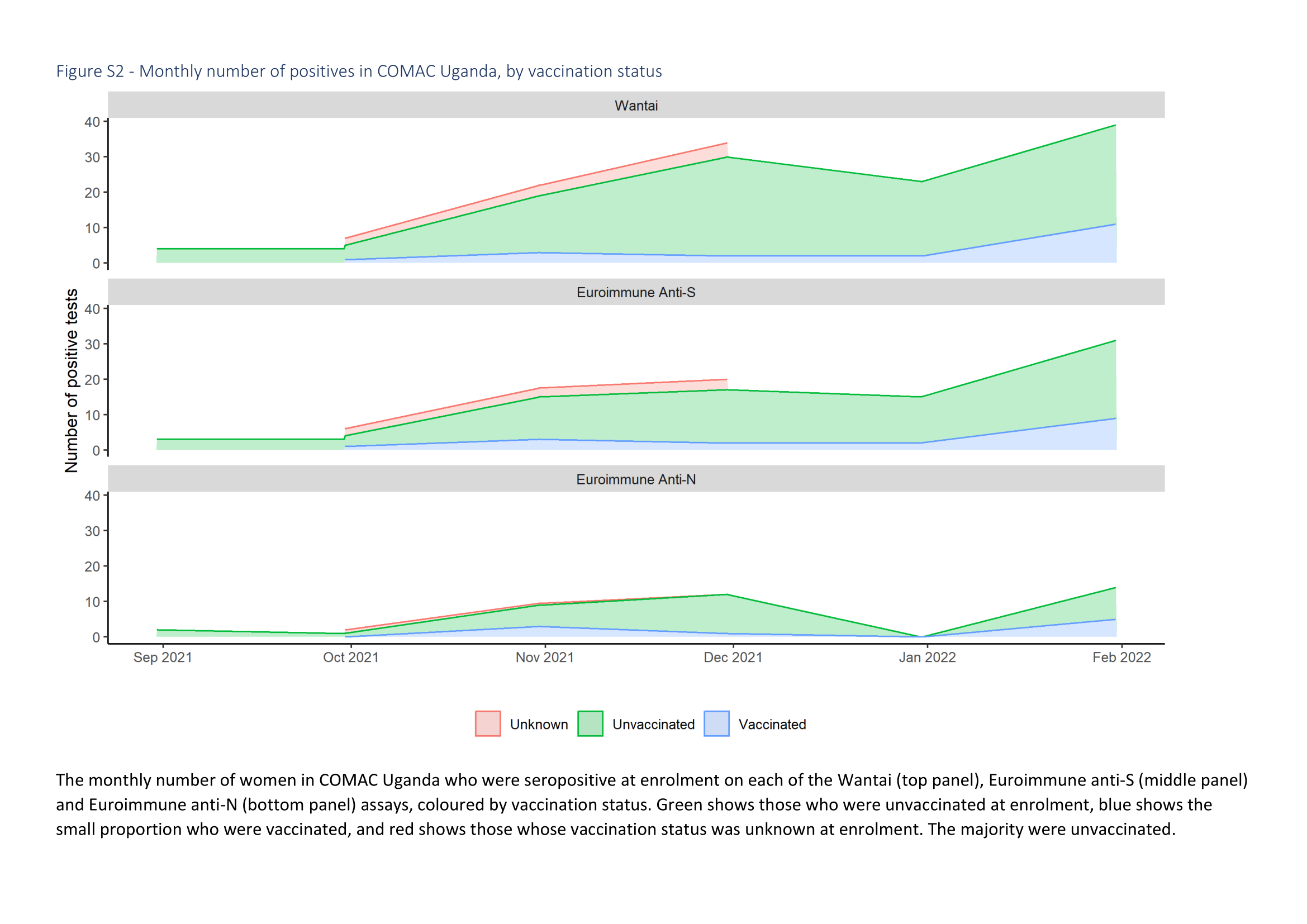

Supplement: S2 Fig — The monthly number of women in COMAC Uganda who were seropositive at enrolment on each of the Wantai (top panel), Euroimmune anti-S (middle panel) and Euroimmune anti-N (bottom panel) assays, coloured by vaccination status. Green shows those who were unvaccinated at enrolment, blue shows the small proportion who were vaccinated, and red shows those whose vaccination status was unknown at enrolment. The majority were unvaccinated. (TIF) [file pone.0290913.s002.tif]
