# Supplementary material for: Seroepidemiology of SARS-CoV-2 in a cohort of pregnant women and their infants in Uganda and Malawi
Source: PLoS One. 2024 Mar 1;19(3):e0290913. doi: 10.1371/journal.pone.0290913 (PMC10906847; doi:10.1371/journal.pone.0290913)
Supplement: S1 Table — Specificity of Wantai assay when tested on pre-COVID samples with and without malaria. (DOCX) [file pone.0290913.s003.docx]

**Table S1. Wantai Assay Specificity**.

|  | Wantai positive (n) | Wantai negative (n) | Specificity (%) |
| --- | --- | --- | --- |
| 2019 samples, malaria positive (n=74) | 1 | 73 | 98.65 |
| 2019 samples, malaria negative (n=100) | 1 | 99 | 99 |
