# Supplementary material for: Seroepidemiology of SARS-CoV-2 in a cohort of pregnant women and their infants in Uganda and Malawi
Source: PLoS One. 2024 Mar 1;19(3):e0290913. doi: 10.1371/journal.pone.0290913 (PMC10906847; doi:10.1371/journal.pone.0290913)
Supplement: S2 Table — Dates used to define the COVID-19 waves, taken from Our World in Data. (DOCX) [file pone.0290913.s004.docx]

**Table S2. Dates used to define COVID-19 waves in Malawi and Uganda**

| Country | Wave 1 | Wave 2 | Wave 3 | Wave 4 |
| --- | --- | --- | --- | --- |
| Uganda | 26/03/2020 – 21/03/2021 | 22/03/2021 – 30/11/2021 | 01/12/2021 – 30/04/2022 |  |
| Malawi | 07/04/2020 – 16/11/2020 | 17/11/2020 – 15/05/2021 | 16/05/2021 – 11/11/2021 | 12/11/2021 – 20/04/2022 |
