# Supplementary material for: Seroepidemiology of SARS-CoV-2 in a cohort of pregnant women and their infants in Uganda and Malawi
Source: PLoS One. 2024 Mar 1;19(3):e0290913. doi: 10.1371/journal.pone.0290913 (PMC10906847; doi:10.1371/journal.pone.0290913)
Supplement: S3 Table — Symptoms are those defined as probable infection according to WHO criteria [18]. (DOCX) [file pone.0290913.s005.docx]

### **Table S3– Maternal PCR tests by study site and wave**

|  | **PeriCOVID Malawi** | | | | **PeriCOVID Uganda** | | |
| --- | --- | --- | --- | --- | --- | --- | --- |
| **Characteristic** | **Overall, N = 73 (95% CI)^12^** | **Wave 2, N = 2 (95% CI)^12^** | **Wave 3, N = 31 (95% CI)^12^** | **Wave 4, N = 40 (95% CI)^12^** | **Overall, N = 836 (95% CI)^12^** | **Wave 1, N = 194 (95% CI)^12^** | **Wave 2, N = 642 (95% CI)^12^** |
| PCR result |  |  |  |  |  |  |  |
| Negative | 24 (33%) (22%, 45%) | 1 (50%) (1.3%, 99%) | 11 (35%) (19%, 55%) | 12 (30%) (17%, 47%) | 817 (98%) (96%, 99%) | 194 (100%) (98%, 100%) | 623 (97%) (95%, 98%) |
| Positive | 49 (67%) (55%, 78%) | 1 (50%) (1.3%, 99%) | 20 (65%) (45%, 81%) | 28 (70%) (53%, 83%) | 19 (2.3%) (1.4%, 3.5%) | 0 (0%) (0.00%, 1.9%) | 19 (3.0%) (1.8%, 4.6%) |
| Symptoms in those with positive PCR |  |  |  |  |  |  |  |
| Asymptomatic | 2 (4.1%) (0.50%, 14%) | 0 (0%) (0.00%, 98%) | 0 (0%) (0.00%, 17%) | 2 (7.1%) (0.88%, 24%) | 13 (68%) (43%, 87%) | NA | 13 (68%) (43%, 87%) |
| Symptomatic | 47 (96%) (86%, 100%) | 1 (100%) (2.5%, 100%) | 20 (100%) (83%, 100%) | 26 (93%) (76%, 99%) | 6 (32%) (13%, 57%) | NA | 6 (32%) (13%, 57%) |

Symptoms are those defined as probable infection according to WHO criteria [18]
