# Supplementary material for: Seroepidemiology of SARS-CoV-2 in a cohort of pregnant women and their infants in Uganda and Malawi
Source: PLoS One. 2024 Mar 1;19(3):e0290913. doi: 10.1371/journal.pone.0290913 (PMC10906847; doi:10.1371/journal.pone.0290913)
Supplement: S4 Table — (DOCX) [file pone.0290913.s006.docx]

**Table S4 – Adverse pregnancy outcomes in mothers enrolled in PeriCOVID Malawi and PeriCOVID Uganda**

|  | **All Enrolled** | **Wantai Overall** | **Wantai Serostatus** | |
| --- | --- | --- | --- | --- |
| **Characteristic** | **N = 1,224^1^** | **N = 1,220^1^** | **Negative, N = 469^2^** | **Positive, N = 751^2^** |
| Maternal death | 4 (0.3%) | 4 (0.3%) | 1 (25%) | 3 (75%) |
| Unknown | 27 | 25 | 9 | 16 |
| Abortion | 4 (0.3%) | 4 (0.3%) | 0 (0%) | 4 (100%) |
| Unknown | 25 | 24 | 9 | 15 |
| Premature labour | 52 (4.3%) | 52 (4.3%) | 20 (38%) | 32 (62%) |
| Unknown | 1 |  |  |  |
| Stillbirth | 26 (2.1%) | 26 (2.1%) | 15 (58%) | 11 (42%) |
| At least one adverse pregnancy outcome | 79 (6.5%) | 79 (6.5%) | 34 (43%) | 45 (57%) |
| No adverse pregnancy outcome | 1,145 (94%) | 1,141 (94%) | 435 (38%) | 706 (62%) |
| Livebirth | 1,198 (98%) | 1,194 (98%) | 454 (38%) | 740 (62%) |

^1^ Column percentages are presented for the overall number of women experiencing each outcome

^2^ Row percentages are presented for the number of seropositive and seronegative women experiencing each outcome
