# Supplementary material for: Seroepidemiology of SARS-CoV-2 in a cohort of pregnant women and their infants in Uganda and Malawi
Source: PLoS One. 2024 Mar 1;19(3):e0290913. doi: 10.1371/journal.pone.0290913 (PMC10906847; doi:10.1371/journal.pone.0290913)
Supplement: S5 Table — (DOCX) [file pone.0290913.s007.docx]

**Table S5- Adverse neonatal outcomes in infants born in PeriCOVID Malawi and PeriCOVID Uganda**

|  | **All infants** | **Wantai Overall** | **Maternal Wantai Serostatus** | |
| --- | --- | --- | --- | --- |
| **Characteristic** | **N = 1,230^1^** | **N = 1,227^1^** | **Negative, N = 474^2^** | **Positive, N = 753^2^** |
| Infant death | 46 (3.8%) | 46 (3.8%) | 20 (43%) | 26 (57%) |
| Unknown | 5 | 4 | 3 | 1 |
| Prematurity | 58 (4.7%) | 58 (4.7%) | 22 (38%) | 36 (62%) |
| Unknown | 1 |  |  |  |
| Low birth weight | 61 (5.0%) | 61 (5.0%) | 22 (36%) | 39 (64%) |
| Unknown | 1 |  |  |  |
| Admitted to NICU | 170 (14%) | 170 (14%) | 63 (37%) | 107 (63%) |
| Unknown | 4 | 3 | 3 | 0 |
| Birth asphyxia | 53 (4.3%) | 53 (4.3%) | 20 (38%) | 33 (62%) |
| Unknown | 1 |  |  |  |
| At least one adverse neonatal outcome | 197 (16%) | 197 (16%) | 77 (39%) | 120 (61%) |
| No adverse neonatal/infant outcome | 1,033 (84%) | 1,030 (84%) | 397 (39%) | 633 (61%) |

^1^ Column percentages are presented for the overall number of infants experiencing each outcome

^2^ Row percentages are presented for the number of infants born to seropositive and seronegative women with each outcome
