# Supplementary material for: Seroepidemiology of SARS-CoV-2 in a cohort of pregnant women and their infants in Uganda and Malawi
Source: PLoS One. 2024 Mar 1;19(3):e0290913. doi: 10.1371/journal.pone.0290913 (PMC10906847; doi:10.1371/journal.pone.0290913)
Supplement: S6 Table — (DOCX) [file pone.0290913.s008.docx]

**Table S6- Impact of infection (seropositive and WHO probable) on key pregnancy and neonatal outcomes**

|  | **Number included in model** | **Number of events** | **Relative Risk** | **95% Confidence Interval** |
| --- | --- | --- | --- | --- |
| **Maternal death** | | | | |
| Infection status |  | 4 |  |  |
| sero-negative |  | 1 | — | — |
| sero-positive and not WHO probable |  | 0 | — | — |
| sero-positive and WHO probable |  | 3 | — | — |
| **Infant death** | | | | |
| Infection status | 1,223 | 46 |  |  |
| sero-negative | 471 | 20 | — | — |
| sero-positive and not WHO probable | 667 | 24 | 0.85 | 0.47, 1.53 |
| sero-positive and WHO probable | 85 | 2 | — | — |
| **Premature labour** | | | | |
| Infection status | 1,234 | 52 |  |  |
| Sero- negative | 476 | 20 | — | — |
| Sero- positive and not WHO probable | 672 | 27 | 0.94 | 0.53, 1.67 |
| Sero- positive and WHO probable | 86 | 5 | 1.24 | 0.42, 3.00 |
| **Still birth** | | | | |
| Infection status | 1,225 | 26 |  |  |
| Sero- negative | 473 | 15 | — | — |
| Sero- positive and not WHO probable | 667 | 10 | 0.48 | 0.21, 1.05 |
| Sero- positive and WHO probable | 85 | 1 | — | — |
| **Abortion** | | | | |
| Infection status |  | 4 |  |  |
| Sero- negative |  | 0 | — | — |
| Sero- positive and not WHO probable |  | 4 | — | — |
| Sero- positive and WHO probable |  | 0 | — | — |
| **Combined adverse pregnancy outcome** | | | | |
| Infection status | 1,205 | 79 |  |  |
| Sero- negative | 463 | 34 | — | — |
| Sero- positive and not WHO probable | 659 | 36 | 0.74 | 0.47, 1.16 |
| Sero- positive and WHO probable | 83 | 9 | 1.39 | 0.64, 2.68 |
| **Low birth weight** | | | | |
| Infection status | 1,227 | 61 |  |  |
| Sero- negative | 474 | 22 | — | — |
| Sero- positive and not WHO probable | 668 | 33 | 0.99 | 0.59, 1.67 |
| Sero- positive and WHO probable | 85 | 6 | 0.98 | 0.37, 2.17 |
| **NICU admission** | | | | |
| Infection status | 1,224 | 170 |  |  |
| Sero- negative | 471 | 63 | — | — |
| Sero- positive and not WHO probable | 668 | 90 | 0.95 | 0.72, 1.28 |
| Sero- positive and WHO probable | 85 | 17 | 1.03 | 0.62, 1.60 |
| **Combined adverse neonatal outcome** | | | | |
| Infection status | 1,234 | 197 |  |  |
| Sero- negative | 476 | 77 | — | — |
| Sero- positive and not WHO probable | 672 | 102 | 0.91 | 0.70, 1.19 |
| Sero- positive and WHO probable | 86 | 18 | 0.98 | 0.60, 1.50 |

Models are adjusted for country
