# Supplementary material for: Seroepidemiology of SARS-CoV-2 in a cohort of pregnant women and their infants in Uganda and Malawi
Source: PLoS One. 2024 Mar 1;19(3):e0290913. doi: 10.1371/journal.pone.0290913 (PMC10906847; doi:10.1371/journal.pone.0290913)
Supplement: S7 Table — (DOCX) [file pone.0290913.s009.docx]

**Table S7- Placental Transfer**

|  | | PeriCOVID Uganda | | COMAC Uganda | |
| --- | --- | --- | --- | --- | --- |
|  |  | Wave 1 | Wave 2 | Wave 2 | Wave 3 |
| Anti-S | Infant, GMC (95% CI) | 78.8 (46.4, 133.7) [N = 27] | 127.6 (100.6, 161.8) [N = 181] | 152 (100.3, 230.3) [N = 60] | 65.2 (47.1, 90.4) [N = 59] |
|  | Mother, GMC (95% CI) | 51.7 (34.6, 77.3) [N = 27] | 74.7 (59.3, 94.2) [N = 181] | 96.1 (65.9, 140.1) [N = 60] | 98.5 (68.6, 141.5) [N = 59] |
|  | Placental transfer, GMR (95% CI) | 1.5 (0.9, 2.5) [N = 27] | 1.7 (1.3, 2.3) [N = 181] | 1.6 (0.8, 3) [N = 60] | 0.7 (0.4, 1) [N = 59] |
| Anti-N | Infant, GMC (95% CI) | 57.1 (38, 85.8) [N = 39] | 133.7 (106.5, 167.8) [N = 194] | 155.9 (102.1, 237.9) [N = 55] | 172 (103, 287.2) [N = 43] |
|  | Mother, GMC (95% CI) | 57.6 (41.5, 79.9) [N = 39] | 146.2 (114.4, 186.8) [N = 194] | 87.6 (54.9, 139.8) [N = 55] | 62.9 (35.5, 111.4) [N = 43] |
|  | Placental transfer, GMR (95% CI) | 1 (0.6, 1.6) [N = 39] | 0.9 (0.7, 1.3) [N = 194] | 1.8 (1.1, 2.9) [N = 55] | 2.7 (1.7, 4.4) [N = 43] |
